# Comparing sodium-glucose cotransporter 2 inhibitors and dipeptidyl peptidase-4 inhibitors on new-onset depression: a propensity score-matched study in Hong Kong

**DOI:** 10.1007/s00592-023-02063-6

**Published:** 2023-03-31

**Authors:** Jonathan V. Mui, Lifang Li, Oscar Hou In Chou, Nida Azfar, Athena Lee, Jeremy Hui, Sharen Lee, Gary Tse, Jiandong Zhou

**Affiliations:** 1 Diabetes Research Unit, Cardiovascular Analytics Group, Hong Kong, China; 2 Department of Biostatistics and Health Informatics, Institute of Psychiatry, Psychology and Neuroscience, King’s College London, London, UK; 3 Department of Medicine, The University of Hong Kong, Hong Kong, China; 4 Department of Medicine & Therapeutics, The Chinese University of Hong Kong, Hong Kong, China; 5 Department of Cardiology, Second Hospital of Tianjin Medical University, Tianjin, China; 6 Kent and Medway Medical School, Canterbury, UK; 7 Nuffield Department of Medicine, University of Oxford, Oxford, UK; 8 School of Nursing and Health Studies, Hong Kong Metropolitan University, Hong Kong, China; 9 Tianjin Institute of Cardiology, The Second Hospital of Tianjin Medical University, Tianjin, 300211 China

**Keywords:** Type 2 diabetes, Depression, Anti-diabetic medication, SGLT2 inhibitor, DPP4 inhibitor

## Abstract

**Introduction:**

The risk of new onset depression associated with sodium-glucose co-transporter 2 inhibitor (SGLT2I) use in patients with type 2 diabetes mellitus (T2DM) remains unclear. This study investigated the risk of new onset depression between SGLT2I and dipeptidyl peptidase 4 inhibitor (DPP4I) users.

**Methods:**

This was a population-based cohort study of T2DM patients in Hong Kong between January 1st, 2015, and December 31st, 2019. T2DM patients over 18 with either SGLT2I or DPP4I use were included. 1:1 propensity-score matching using the nearest-neighbour method was conducted based on demographics, past comorbidities and non-DPP4I/SGLT2I medication use. Cox regression analysis models were used to identify significant predictors for new onset depression.

**Results:**

The study cohort included a total of 18,309 SGLT2I users and 37,269 DPP4I users (55.57% male, mean age: 63.5 ± 12.9 years) with a median follow-up duration of 5.56 (IQR: 5.23–5.8) years. After propensity score matching, SGLT2I use was associated with a lower risk of new onset depression compared to DPP4I use (HR: 0.52, 95% CI: [0.35, 0.77], *P* = 0.0011). These findings were confirmed by Cox multivariable analysis and sensitive analyses.

**Conclusion:**

SGLT2I use is associated with significantly lower risk of depression compared to DPP4 use in T2DM patients using propensity score matching and Cox regression analyses.

**Supplementary Information:**

The online version contains supplementary material available at 10.1007/s00592-023-02063-6.

## Introduction

Type 2 diabetes mellitus (T2DM) has been described as an emerging pandemic, currently affecting over 462 million people worldwide [1]. Equally alarming is the recent increase in prevalence of depression especially during the COVID pandemic, a common mental health disorder which affects approximately 280 million people worldwide [2, 3]. A close link between the two conditions has long been recognised since the seventeenth century, with the famous British physician Thomas Phyllis describing diabetes as “a consequence of prolonged sorrow” [4]. This link has been confirmed by various studies demonstrating that there is an increased prevalence and diagnosis of depression in T2DM patients [5, 6] with one study reporting that T2DM doubles the risk of depression [5]. Conversely, it has also been shown that depression increases the risk of developing diabetes [7, 8] and diabetic complications [9, 10], indicating a bi-directional relationship between the two.

Given the close relationship between diabetes and depression, there has been growing interest to study the modulatory effects of anti-diabetic medications on depression, including novel agents such as dipeptidyl peptidase 4 inhibitor (DPP4I) and sodium-glucose co-transporter 2 inhibitor (SGLT2I). While early case reports suggested a potential association between incretin-based therapies and depression [11, 12], recent cohort studies have found that DPP4I use is generally associated with a reduced risk of depression [13–16]. This is confirmed by clinical findings that DPP4 enzymatic activity is increased in patients with depressive symptoms [17] as well as pre-clinical findings in rodent model that DPP4I use produces antidepressant effects [18, 19]. By contrast, there is limited data available to assess the anti-depressant effects of SGLT2I. A cohort study in 2019 found that both DPP4I and SGLT2I were associated with significantly lower risk of depression, but was only based on 1 SGLT2I user [13]. A case report in 2020 described a patient whose depressive symptoms and suicidal ideations resolved after 1 year of SGLT2I initiation [20]. While both showed promising results, it has not been possible to draw any definitive conclusions due to the small sample size of SGLT2I users in the respective studies.

To our knowledge, there has been no large-scale study so far exploring SGLT2I and its association with depression, either in isolation or in a head-to-head comparison with DPP4I. Hence, the aim of this study is to explore the largely unknown association with depression of SGLT2I use as compared against DPP4I, using a large database of Chinese T2DM patients in Hong Kong.

## Methods

### Ethics approval

This study was approved by the Joint Chinese University of Hong Kong–New Territories East Cluster Clinical Research Ethics Committee (Ethics Committee Approval Number NTEC-2018-0563).

### Data sources and study population

**Fig. 1:**
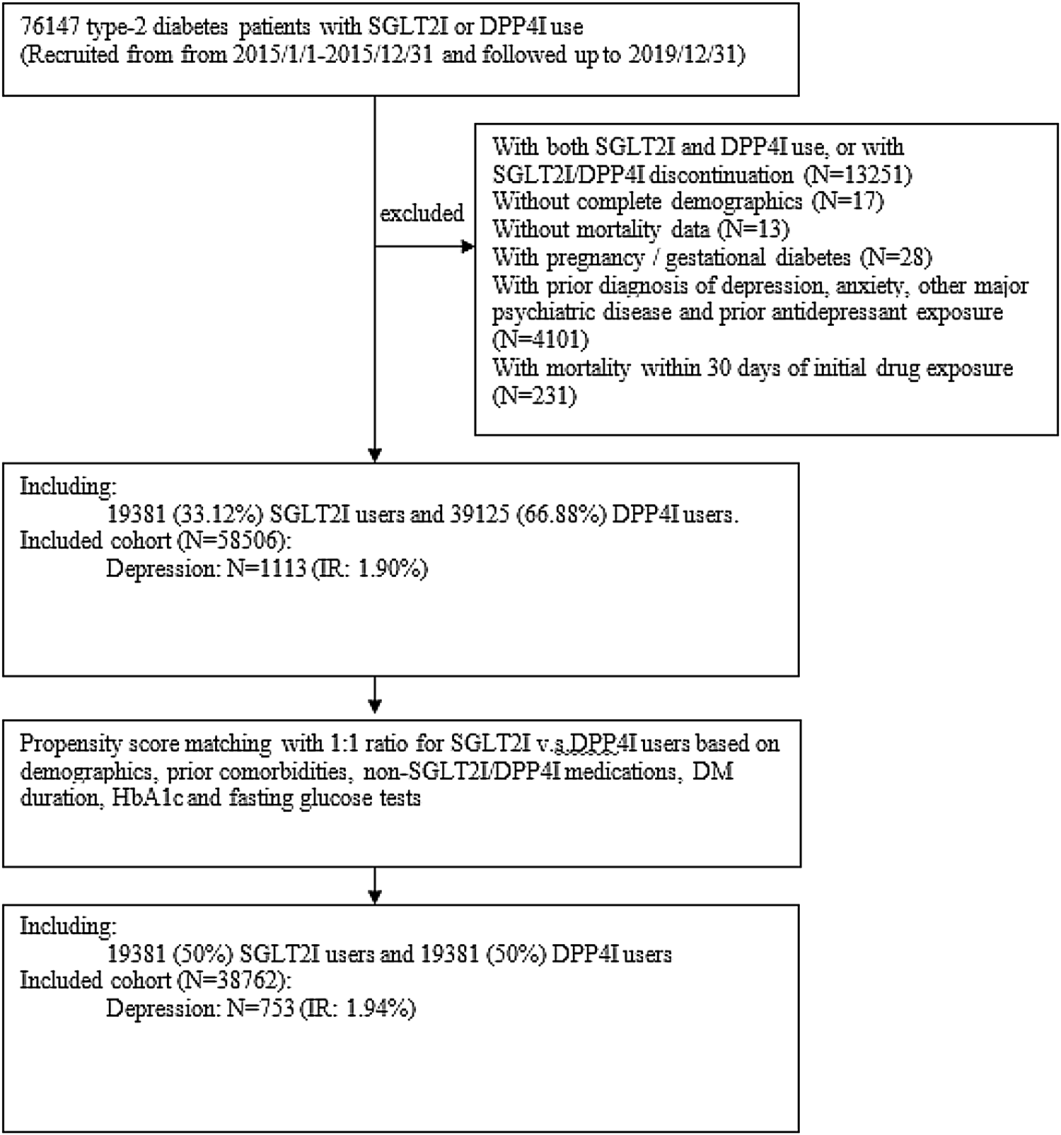
Procedures of data processing for the study cohort. IR: Incidence rate; SGLT2I: Sodium-glucose cotransporter-2 inhibitors; DPP4I: Dipeptidyl peptidase-4 inhibitors

This was a retrospective, territory-wide cohort study of T2DM patients in Hong Kong with SGLT2I/DPP4I use between January 1st, 2015, and December 31st, 2019 (Fig. 1). Patients during the aforementioned study period were enrolled and followed up until December 31st, 2019 or until death. Our team has previously used this large dataset for investigating outcomes including atrial fibrillation, stroke, myocardial infarction, heart failure and dementia [21–23].

The patients were identified from the Clinical Data Analysis and Reporting System (CDARS), a territory-wide database that centralizes patient information from individual local hospitals to establish a comprehensive set of medical data, including clinical characteristics, disease diagnosis, laboratory results, and drug treatment details. The system has previously been used by both our team and other teams in Hong Kong to conduct epidemiological studies [24–26].

As SGLT2I and DPP4I were only licensed for use in Hong Kong from 2015 onwards, the study is effectively a new user design with all users starting use of the medication during the study period. Patients were followed up from their first use of the medication either until the diagnosis of new-onset depression or until death. Certain patients were excluded from the study cohort, including patients with both DPP4I and SGLT2I use or discontinuation during the study period, without complete demographics data, without mortality data, with pregnancy or gestational diabetes and with prior diagnosis of psychiatric disease of antidepressant exposure. Users of both DPP4I and SGLT2I were excluded to ascertain the effects were due to one of the drugs, as it would be difficult to attribute whether the risk of new-onset depression was due to DPP4I use, SGLT2I use, or a combination of both with or without switching drugs. As drug compliance is not routinely collected within CDARs, users’ compliance to medication is only assessed indirectly through prescription refills.

Clinical and biochemical data were extracted for the present study. Patients' demographics included gender and age of initial drug use (baseline). Prior comorbidities before initial drug use were extracted based on standard *International Classification of Diseases Ninth Edition* (ICD-9) codes as shown in Supplementary Table 1 and the Charlson comorbidity index was also calculated. Baseline anti-5diabetic medication use, including metformin, sulphonylurea, insulin, acarbose, thiazolidinedione and glucagon-like peptide-1 agonist, was extracted. Baseline laboratory data were also extracted, including complete blood count, biochemical tests, glucose and lipid profiles.

### Statistical analysis

Descriptive statistics were used to summarize baseline characteristics of patients with SGLT2I and DPP4I use. For baseline clinical characteristics, the continuous variables were presented as mean (standard deviation [SD]) or median (95% confidence interval [CI]/ interquartile range [IQR]), and the categorical variables were presented as total number (percentage). Continuous variables were compared using the two-tailed Mann-Whitney U test, whilst the two-tailed Chi-square test with Yates’ correction was used to test 2 × 2 contingency data. 1:1 propensity score matching between SGLT2I and DPP4I users was performed based on demographics, prior comorbidities and non-SGLT2I/DPP4I medication using the nearest neighbour search strategy with calliper of 0.1. Propensity score matching results between treatment-group (SGLT2I) versus control-group (DPP4I) before and after matching are shown in Supplementary Fig. 1.

Univariate and multivariable Cox regression models were used to identify significant risk predictors for the study outcomes. Regression analysis with one-year lag time, competing risk analysis (cause-specific and sub-distribution models) and different propensity score approaches (propensity score stratification [27], propensity score matching with inverse probability weighting [28] and propensity score matching with stable inverse probability weighting [29]) were also considered. The hazard ratio (HR), 95% CI and P-value were reported. Statistical significance was defined as *P*-value < 0.05. All statistical analyses were performed with RStudio software (Version: 1.1.456), Python (Version: 3.6), and Stata (Version: SE 16.0).

## Results

### Baseline characteristics before and after propensity score matching

From the 76,147 patients identified on CDARS within the study period, we excluded 17,641 patients including patients with both DPP4I and SGLT2I use or discontinuation during the study period (N = 13,251), without complete demographics data (N = 17), without mortality data (N = 13), with pregnancy or gestational diabetes (N = 28), with prior diagnosis of psychiatric disease of antidepressant exposure (N = 4101) and with mortality within 30 days of initial drug exposure (N = 231).

After exclusion, the study cohort included 58,506 patients comprising of 19,381 SGLT2I users and 39,125 DPP4I users. The median age was 63.4 years old and 56.10% were male. After a median follow-up duration of 5.56 years [IQR: 5.23–5.8], 1113 (1.90%) patients developed new onset depression including 200(0.34%) SGLT2I users and 913(1.56%) DPP4I users. After 1:1 propensity score matching, the study cohort included 38,762 patients comprising of 19,381 SGLT2I users and 19,381 DPP4I users. 753 (1.94%) patients developed new onset depression including 200 (0.52%) SGLT2I users and 553 (1.43%) DPP4I users. The baseline and clinical characteristics of DPP4I and SGLT2I users before and after propensity score matching are summarized in Table 1. After propensity score matching, most variables showed standardised mean difference (SMD) < 0.2, indicating successful matching. The basic and clinical characteristics of patients with/without new onset depression before and after propensity score matching are summarised in Supplementary Table 2.

**Table 1:** Baseline and clinical characteristics of patients with DPP4I v.s. SGLT2I use before and after propensity score matching (1:1)

Characteristics	Before matching			Standardised mean difference (SMD)^#^	After matching			Standardised mean difference (SMD)^#^
	All (N = 58,506) Mean(SD);N or Count(%)	SGLT2I users (N = 19,381) Mean(SD);N or Count(%)	DPP4I users (N = 39,125) Mean(SD);N or Count(%)		All (N = 38,762) Mean(SD);N or Count(%)	SGLT2I users (N = 19,381) Mean(SD);N or Count(%)	DPP4I users (N = 19,381) Mean(SD);N or Count(%)	
*Demographics*								
Male gender	32,825(56.10%)	12,007(61.95%)	20,818(53.20%)	0.18	24,102(62.17%)	12,007(61.95%)	12,095(62.40%)	0.01
Female gender	25,681(43.89%)	7374(38.04%)	18,307(46.79%)	0.18	14,660(37.82%)	7374(38.04%)	7286(37.59%)	0.01
Baseline age, years	63.4(12.9);n = 58,506	57.7(11.2);n = 19,381	66.2(12.7);n = 39,125	0.71*	58.1(11.2);n = 38,762	57.7(11.2);n = 19,381	58.5(11.2);n = 19,381	0.07
Diabetes duration, days	500.7(1314.4);n = 58,506	499.6(1160.4);n = 19,381	501.2(1384.3);n = 39,125	< 0.01	459.8(1188.6);n = 38,762	499.6(1160.4);n = 19,381	420.0(1214.8);n = 19,381	0.07
*Past comorbidities*								
Charlson comorbidity index	2.1(1.5);n = 58,506	1.6(1.2);n = 19,381	2.4(1.6);n = 39,125	0.61*	1.6(1.2);n = 38,762	1.55(1.24);n = 19,381	1.56(1.24);n = 19,381	0.01
Heart failure	1939(3.31%)	490(2.52%)	1449(3.70%)	0.07	968(2.49%)	490(2.52%)	478(2.46%)	< 0.01
Hypertension	13,924(23.79%)	4519(23.31%)	9405(24.03%)	0.02	8865(22.87%)	4519(23.31%)	4346(22.42%)	0.02
Hypoglycaemia	476(0.81%)	45(0.23%)	431(1.10%)	0.11	90(0.23%)	45(0.23%)	45(0.23%)	< 0.01
Hyperlipidaemia	1559(2.66%)	682(3.51%)	877(2.24%)	0.08	1344(3.46%)	682(3.51%)	662(3.41%)	0.01
Ischemic heart disease	5823(9.95%)	2502(12.90%)	3321(8.48%)	0.14	4610(11.89%)	2502(12.90%)	2108(10.87%)	0.06
Liver diseases	2210(3.77%)	904(4.66%)	1306(3.33%)	0.07	1746(4.50%)	904(4.66%)	842(4.34%)	0.02
Autoimmune disease tissue	591(1.01%)	189(0.97%)	402(1.02%)	0.01	377(0.97%)	189(0.97%)	188(0.97%)	< 0.01
Gastrointestinal disease	1378(2.35%)	347(1.79%)	1031(2.63%)	0.06	688(1.77%)	347(1.79%)	341(1.75%)	< 0.01
Acute myocardial infarction	1598(2.73%)	660(3.40%)	938(2.39%)	0.06	1311(3.38%)	660(3.40%)	651(3.35%)	< 0.01
Peripheral vascular disease	455(0.77%)	101(0.52%)	354(0.90%)	0.05	202(0.52%)	101(0.52%)	101(0.52%)	< 0.01
Chronic obstructive pulmonary disease	684(1.16%)	139(0.71%)	545(1.39%)	0.07	278(0.71%)	139(0.71%)	139(0.71%)	< 0.01
Renal diseases	1152(1.96%)	102(0.52%)	1050(2.68%)	0.17	204(0.52%)	102(0.52%)	102(0.52%)	< 0.01
Sleep disorders	1749(2.98%)	1005(5.18%)	744(1.90%)	0.18	1908(4.92%)	1005(5.18%)	903(4.65%)	0.02
Stroke/transient ischemic attack	1854(3.16%)	489(2.52%)	1365(3.48%)	0.06	970(2.50%)	489(2.52%)	481(2.48%)	< 0.01
Atrial fibrillation	1523(2.60%)	426(2.19%)	1097(2.80%)	0.04	845(2.17%)	426(2.19%)	419(2.16%)	< 0.01
Anaemia	2462(4.20%)	423(2.18%)	2039(5.21%)	0.16	847(2.18%)	423(2.18%)	424(2.18%)	< 0.01
Cancer	1629(2.78%)	391(2.01%)	1238(3.16%)	0.07	780(2.01%)	391(2.01%)	389(2.00%)	< 0.01
*Medications*								
Metformin	51,824(88.57%)	18,016(92.95%)	33,808(86.41%)	0.22*	36,057(93.02%)	18,016(92.95%)	18,041(93.08%)	0.01
Sulphonylurea	44,983(76.88%)	13,618(70.26%)	31,365(80.16%)	0.23*	27,844(71.83%)	13,618(70.26%)	14,226(73.40%)	0.07
Insulin	29,437(50.31%)	9829(50.71%)	19,608(50.11%)	0.01	20,695(53.38%)	9829(50.71%)	10,866(56.06%)	0.11
Acarbose	1470(2.51%)	778(4.01%)	692(1.76%)	0.13	1455(3.75%)	778(4.01%)	677(3.49%)	0.03
Thiazolidinedione	10,758(18.38%)	5330(27.50%)	5428(13.87%)	0.34*	9524(24.57%)	5330(27.50%)	4194(21.63%)	0.14
Glucagon-like peptide-1 receptor agonists	1572(2.68%)	1407(7.25%)	165(0.42%)	0.36*	2432(6.27%)	1407(7.25%)	1025(5.28%)	0.08
Statins and fibrates	15,292(26.13%)	2344(12.09%)	12,948(33.09%)	0.52*	4517(11.65%)	2344(12.09%)	2173(11.21%)	0.03
ACEI/ARB	12,420(21.22%)	8089(41.73%)	4331(11.06%)	0.74*	15,588(40.21%)	8089(41.73%)	7499(38.69%)	0.06
Antihypertensive drugs	1018(1.73%)	943(4.86%)	75(0.19%)	0.30*	1227(3.16%)	943(4.86%)	284(1.46%)	0.2
Anticoagulants	17,378(29.70%)	11,514(59.40%)	5864(14.98%)	1.03*	23,078(59.53%)	11,514(59.40%)	11,564(59.66%)	0.01
Antiplatelets	9154(15.64%)	6272(32.36%)	2882(7.36%)	0.66*	11,252(29.02%)	6272(32.36%)	4980(25.69%)	0.15
Lipid-lowering drugs	10,999(18.79%)	6624(34.17%)	4375(11.18%)	0.57*	13,875(35.79%)	6624(34.17%)	7251(37.41%)	0.07
Nitrates	4239(7.24%)	2861(14.76%)	1378(3.52%)	0.40*	5212(13.44%)	2861(14.76%)	2351(12.13%)	0.08
Non-steroidal anti-inflammatory drugs	8796(15.03%)	6020(31.06%)	2776(7.09%)	0.64*	10,950(28.24%)	6020(31.06%)	4930(25.43%)	0.13
Diuretics	9467(16.18%)	6014(31.03%)	3453(8.82%)	0.58*	10,857(28.00%)	6014(31.03%)	4843(24.98%)	0.13
Beta-blockers	7293(12.46%)	5016(25.88%)	2277(5.81%)	0.57*	9044(23.33%)	5016(25.88%)	4028(20.78%)	0.12
Calcium channel blockers	12,606(21.54%)	8458(43.64%)	4148(10.60%)	0.80*	15,173(39.14%)	8458(43.64%)	6715(34.64%)	0.19
*Complete blood counts*								
Mean corpuscular volume, fL	87.2(7.6);n = 29,704	86.7(7.2);n = 10,965	87.6(7.8);n = 18,739	0.12	86.7(7.4);n = 21,160	86.7(7.2);n = 10,965	86.8(7.7);n = 10,195	0.01
Eosinophil, × 10^9/L	0.2(0.3);n = 23,916	0.22(0.2);n = 8569	0.22(0.28);n = 15,347	< 0.01	0.2(0.2);n = 17,345	0.22(0.2);n = 8569	0.23(0.28);n = 8776	0.05
Lymphocyte, × 10^9/L	2.0(0.9);n = 23,941	2.2(0.9);n = 8574	1.9(0.9);n = 15,367	0.32*	2.1(0.9);n = 17,353	2.2(0.9);n = 8574	2.0(0.9);n = 8779	0.17
Neutrophil, × 10^9/L	5.4(2.8);n = 23,941	5.1(2.4);n = 8574	5.5(3.1);n = 15,367	0.14	5.3(2.8);n = 17,353	5.1(2.4);n = 8574	5.5(3.2);n = 8779	0.12
White cell count, × 10^9/L	8.0(3.0);n = 29,713	7.97(2.58);n = 10,971	8.04(3.23);n = 18,742	0.02	8.1(3.1);n = 21,177	8.0(2.6);n = 10,971	8.1(3.5);n = 10,206	0.06
Mean cell haemoglobin, pg	29.4(3.0);n = 29,704	29.2(2.9);n = 10,965	29.6(3.1);n = 18,739	0.15	29.2(3.0);n = 21,160	29.2(2.9);n = 10,965	29.3(3.2);n = 10,195	0.06
Platelet, × 10^9/L	239.3(72.4);n = 29,711	244.1(66.9);n = 10,969	236.4(75.3);n = 18,742	0.11	245.6(72.6);n = 21,175	244.1(66.9);n = 10,969	247.1(78.2);n = 10,206	0.04
Red cell count, × 10^12/L	4.5(0.7);n = 29,704	4.8(0.6);n = 10,965	4.4(0.7);n = 18,739	0.61*	4.7(0.7);n = 21,160	4.8(0.6);n = 10,965	4.6(0.7);n = 10,195	0.28*
*Liver and renal functions*								
Potassium, mmol/L	4.4(0.5);n = 48,394	4.3(0.4);n = 16,344	4.4(0.5);n = 32,050	0.14	4.3(0.5);n = 31,796	4.31(0.43);n = 16,344	4.31(0.49);n = 15,452	0.01
Albumin, g/L	41.5(4.0);n = 37,036	42.5(3.3);n = 13,865	40.9(4.3);n = 23,171	0.41*	42.1(3.8);n = 26,483	42.5(3.3);n = 13,865	41.6(4.3);n = 12,618	0.24*
Sodium, mmol/L	139.3(3.0);n = 48,419	139.2(2.7);n = 16,346	139.3(3.1);n = 32,073	0.04	139.3(2.9);n = 31,824	139.2(2.7);n = 16,346	139.4(3.0);n = 15,478	0.06
Urea, mmol/L	6.7(3.7);n = 48,403	5.7(2.0);n = 16,340	7.2(4.2);n = 32,063	0.45*	6.2(2.9);n = 31,829	5.7(2.0);n = 16,340	6.7(3.5);n = 15,489	0.33*
Protein, g/L	73.8(5.6);n = 34,805	74.4(4.9);n = 13,072	73.4(5.9);n = 21,733	0.18	74.3(5.4);n = 25,214	74.4(4.9);n = 13,072	74.2(6.0);n = 12,142	0.03
Creatinine, umol/L	97.2(80.2);n = 48,545	79.1(28.0);n = 16,375	106.4(95.1);n = 32,170	0.39*	87.8(53.9);n = 31,883	79.1(28.0);n = 16,375	97.1(70.5);n = 15,508	0.34*
Alkaline phosphatase, U/L	77.2(32.9);n = 37,156	73.5(26.2);n = 13,869	79.5(36.2);n = 23,287	0.19	76.6(30.6);n = 26,541	73.5(26.2);n = 13,869	80.1(34.4);n = 12,672	0.21*
Aspartate transaminase, U/L	28.2(54.7);n = 14,759	28.4(29.0);n = 5574	28.1(65.5);n = 9185	0.01	29.4(33.4);n = 11,266	28.4(29.0);n = 5574	30.4(37.3);n = 5692	0.06
Alanine transaminase, U/L	28.7(34.3);n = 31,584	32.3(28.3);n = 11,792	26.6(37.3);n = 19,792	0.17	31.5(28.3);n = 22,033	32.3(28.3);n = 11,792	30.5(28.3);n = 10,241	0.06
Bilirubin, umol/L	11.2(6.9);n = 36,964	11.5(5.6);n = 13,837	11.1(7.6);n = 23,127	0.06	11.2(6.0);n = 26,437	11.5(5.6);n = 13,837	10.9(6.4);n = 12,600	0.09
*Lipid and glucose profiles*								
Triglyceride, mmol/L	1.7(1.5);n = 45,525	1.8(1.7);n = 15,707	1.6(1.3);n = 29,818	0.09	1.8(1.6);n = 30,166	1.79(1.74);n = 15,707	1.81(1.53);n = 14,459	0.01
Low-density lipoprotein, mmol/L	2.4(0.8);n = 44,805	2.37(0.8);n = 15,458	2.38(0.8);n = 29,347	< 0.01	2.4(0.8);n = 29,638	2.37(0.8);n = 15,458	2.42(0.85);n = 14,180	0.06
High-density lipoprotein, mmol/L	1.2(0.3);n = 45,465	1.16(0.31);n = 15,684	1.22(0.34);n = 29,781	0.16	1.2(0.3);n = 30,116	1.16(0.31);n = 15,684	1.19(0.36);n = 14,432	0.1
Total cholesterol, mmol/L	4.3(1.0);n = 45,570	4.31(1.0);n = 15,727	4.32(0.98);n = 29,843	0.01	4.4(1.0);n = 30,192	4.3(1.0);n = 15,727	4.4(1.0);n = 14,465	0.09
Haemoglobin A1C, %	8.0(1.5);n = 47,584	8.3(1.6);n = 16,132	7.9(1.5);n = 31,452	0.24*	8.2(1.6);n = 31,318	8.3(1.6);n = 16,132	8.1(1.7);n = 15,186	0.1
Fasting glucose, mmol/L.1	8.9(3.9);n = 43,006	9.2(3.6);n = 14,806	8.7(4.0);n = 28,200	0.11	9.2(4.4);n = 28,132	9.15(3.59);n = 14,806	9.19(5.12);n = 13,326	0.01

^*^for (standardised mean difference) SMD $\ge$ 0.2; SD: standard deviation; SGLT2I: sodium glucose cotransporter-2 inhibitor; DPP4I: dipeptidyl peptidase-4 inhibitor; # indicates characteristics differences between SGLT2I users and DPP4I users

### Univariate and multivariable cox regression analyses

**Fig. 2:**
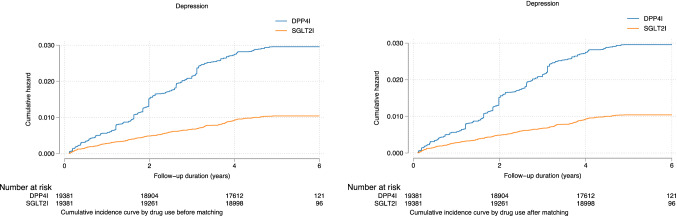
Cumulative incidence curves for new onset depression by SGLT2I vs DPP4I use before and after propensity score matching (1:1)

Univariate Cox regression models were conducted to identify significant predictors of new-onset depression after 1:1 propensity score matching, as presented in Supplementary Table 3. Compared to DPP4I, SGLT2I use was associated with significantly lower incidence of new onset depression both before (HR: 0.42, 95% CI: [0.36, 0.49], *P* < 0.0001) and after matching (HR: 0.35, 95% CI: [0.30, 0.41], *P* < 0.0001). Different multivariable Cox regression models adjusting for significant demographics, past co-morbidities, non-SGLT2I/DPP4I medications, HbA1c, fasting glucose and duration of diabetes were performed as presented in Table 2. SGLT2I continued to demonstrate significantly lower association with new onset depression compared to DPP4I after adjusting for the above (HR: 0.33, 95% CI: [0.27, 0.77], *P* < 0.0001). The cumulative incidence curves for new onset depression in DPP4I and SGLT2I users before and after propensity score matching are presented in Fig. 2.

**Table 2:** Multivariable Cox analysis for new onset depression in the matched cohort

Characteristics	N or count (%)	Model 1	Model 2	Model 3
		Depression HR [95% CI];P value	Depression HR [95% CI];P value	Depression HR [95% CI];P value
SGLT2I.v.s. DPP4I	19,381(50.00%)	0.35[0.30–0.41]; < 0.0001***	0.35[0.29–0.41]; < 0.0001***	0.33[0.27–0.77]; < 0.0001***
Dapagliflozin v.s. DPP4I	11,169(28.81%)	0.47[0.39–0.58]; < 0.0001***	0.44[0.32–0.67]; < 0.0001***	0.44[0.33–0.86]; < 0.0001***
Empagliflozin v.s. DPP4I	4286(11.05%)	0.44[0.32–0.61]; < 0.0001***	0.51[0.26–0.81]; < 0.0001***	0.45[0.30–0.90]; < 0.0001***
Canagliflozin v.s. DPP4I	4667(12.04%)	0.34[0.24–0.49]; < 0.0001***	0.36[0.21–0.59]; < 0.0001***	0.39[0.20–0.83]; < 0.0001***
Ertugliflozin v.s. DPP4I	2367(6.10%)	0.34[0.21–0.57]; < 0.0001***	0.39[0.31–0.77]; < 0.0001***	0.36[0.19–0.82]; < 0.0001***

^*^p ≤ 0.05, **p ≤ 0.01, ***p ≤ 0.001; HR: hazard ratio; CI: confidence interval; SGLT2I: sodium glucose cotransporter-2 inhibitor; DPP4I: dipeptidyl peptidase-4 inhibitor

Model 1 adjusted for significant demographics

Model 2 adjusted for significant demographics, and past comorbidities

Model 3 adjusted for significant demographics, past comorbidities, non-SGLT2I/DPP4I medications, HbA1c, fasting glucose, and duration from diabetes diagnosis to drug exposure

### Sensitivity analysis

Finally, sensitivity analyses for the effects of SGLT2I versus DPP4I use on new onset depression were conducted as presented in Table 3. These included regression analysis with one-year lag time, competing risk analyses using cause-specific and sub-distribution hazard models on the matched cohorts and different propensity score-based approaches on the cohort. These analyses confirmed the same findings from the Cox regression models that SGLT2I use is associated with significantly lower incidence of new-onset depression compared to DPP4I use.

**Table 3:** Sensitivity analyses for SGLT2I v.s. DPP4I exposure effects on new onset depression in the matched cohort

Model	New onset depression
Regression analysis with one-year lag time	0.34[0.21–0.65];0.0004***
Cause-specific hazard model	0.43[0.32–0.85]; < 0.0001***
Sub-distribution hazard model	0.62[0.33–0.77]; < 0.0001***
PS stratification	0.38[0.24–0.73]; < 0.0001***
PS with IPTW	0.49[0.34–0.88];0.0015**
PS with SIPTW	0.58[0.44–0.91];0.0033**

*p ≤ 0.05, **p ≤ 0.01, ***p ≤ 0.001; SGLT2I: Sodium-glucose cotransporter-2 inhibitors; DPP4I: Dipeptidyl peptidase-4 inhibitors; HR: hazard ratio; CI: confidence interval; PS: propensity score; IPTW: inverse probability of treatment weighting, SIPTW: stable inverse probability of treatment weighting

## Discussion

This key finding of the present study is that SGLT2I users are associated with a lower risk of depression compared to DPP4I users after 1:1 propensity score matching for demographics, prior comorbidities, non-SGLT2I/DPP4I medication use, glycaemic indices and duration of diabetes. This was demonstrated by Cox regression models and further confirmed by competing risk analysis and different propensity score approaches.

Several studies have previously shown that the risk of depression is significantly lowered by DPP4I use in T2DM patients. A prospective study in 2016 of 1735 T2DM patients found that one year of incretin-based therapy use, defined as glucagon-like peptide-1 receptor agonist (GLP1-RA) or DPP4I, was correlated with significant improvement in depressive symptoms as measured by the Patient Health Questionnaire-9 [15]. A UK cohort study in 2018 found that DPP4I use is associated with a lower risk of new-onset depression and self-harm compared to sulphonylurea (HR: 0.80, 95% CI: [0.57, 1.13]) but did not reach statistical threshold [14]. A Japanese cohort study in 2019 of 40,214 patients investigated all classes of anti-diabetic medications and found that only DPP4I use was associated with significantly lower risk for development of depression (HR: 0.31, 95% CI: [0.24, 0.42], *P* < 0.0001) [13]. This has also been confirmed in animal models, such as a study in 2016 demonstrating that sitagliptin has anti-nociceptive and antidepressant effects using a rodent model of depression [18]. Compared to DPP4I, research on the association between SGLT2I and depression has been very limited. The aforementioned 2019 Japanese study is the only study to-date to investigate the association between SGLT2I use and depression [13]. The study suggested that SGLT2I use significantly reduces the risk of depression (HR: 0.09, 95% CI: [0.01–0.63], *P* = 0.0153), but was only based on 1 SGLT2I patient and therefore inconclusive.

Multiple studies have demonstrated the neuroprotective effects of SGLT2I, highlighting their potential to improve brain mitochondrial function, hippocampal synaptic plasticity and inhibit acetylcholinesterase [30–33]. It is therefore very possible that SGLT2I exerts its anti-depressant effects via direct effects on the brain. One such mechanism was suggested in a recent study by Muhammad et al. using a rodent model of depression [34]. The neuroimmune hypothesis of depression suggests that mood disorders are mediated by a state of systemic inflammation, defined by activated inflammatory pathways and elevated cytokine levels [35–37]. One such pathway is the nod-like receptor pyrin containing 3 (NLRP3) inflammasome which, when activated in chronic stress, leads to release of pro-inflammatory cytokines such as IL-1β and IL-18 [38]. Muhammad et al. demonstrated that dapagliflozin suppresses NLRP3 inflammasome activation and downstream inflammatory mediators, thus inhibiting neuro-inflammation and blood-brain barrier disturbances. The study also demonstrated that the mechanism of action and efficacy shown by dapagliflozin was analogous, and sometimes superior, to the commonly prescribed anti-depressant Escitalopram [34]. While further studies are required to confirm whether such effects are observed in humans, it gives credence to the exciting anti-depressant potentials of SGLT2I in addition to its main anti-diabetic effects among T2DM patients.

### Limitations

Several limitations should be noted for the present study. First, given its observational nature, there is inherent information bias due to under-coding, coding errors and missing data. Secondly, as drug compliance is not routinely collected within CDARS, patient compliance to SGLT2I and DPP4I was only assessed indirectly through prescription refills and was not accounted for in Cox regression analyses. Thirdly, residual and unmeasured confounding may be present despite robust propensity-matching, particularly with the unavailability of information such as patient-level socioeconomic status. Patients’ drug exposure duration has not been controlled, which may affect their risk against the study outcomes.

## Conclusion

SGLT2I use is associated with significantly lower risk of depression compared to DPP4 use in patients with type-2 diabetes mellitus using propensity score matching and Cox regression analyses.

## Supplementary Information

Below is the link to the electronic supplementary material.

Supplementary file1 (DOCX 93 KB)
