# Supplementary material for: Comparing sodium-glucose cotransporter 2 inhibitors and dipeptidyl peptidase-4 inhibitors on new-onset depression: a propensity score-matched study in Hong Kong
Source: Acta Diabetol. 2023 Mar 31;60(7):917–27. doi: 10.1007/s00592-023-02063-6 (PMC10198893; doi:10.1007/s00592-023-02063-6)
Supplement: Supplementary file 1 — Supplementary file1 (DOCX 93 KB) [file 592_2023_2063_MOESM1_ESM.docx]

**Supplementary Figure 1. Propensity score matching for SGLT2I use versus DPP4I use before and after 1:1 matching with nearest neighbor search strategy with with caliper of 0.1.**

**Supplementary Table 1. ICD9 codes for comorbidities and ICD10 codes for outcomes.**

Depression 296.2 296.22 296.23 296.3 296.32 296.33 311

Anxiety disorder 300 300.01 300.02 300.09 300.21 300.22 300.23 300.29 300.3 300.4 300.6 300.7 300.81 300.82 300.89 300.9

Heart failure 428 428.1 428.2 428.21 428.22 428.23 428.3 428.31 428.32 428.33 428.4 428.41 428.42 428.43 428.9 398.91 402.01 402.11 402.91 404.01 404.03 404.11 404.13 404.91 404.93

Hypertension 401 401.1 401.9 402 402.01 402.1 402.11 402.9 402.91 403 403.01 403.1 403.11 403.9 403.91 404 404.01 404.02 404.03 404.1 404.11 404.12 404.13 404.9 404.91

Hypoglycemia 251.2

Hyperlipidaemia 272 272 272.1 272.2 272.3

Ischemic heart disease 410.01 410.02 410.1 410.11 410.12 410.2 410.21 410.22 410.3 410.31 410.32 410.4 410.41 410.42 410.5 410.51 410.52 410.6 410.61 410.62 410.7 410.71 410.72 410.8 410.81

Liver diseases 456 456.1 456.2 572.2 572.3 572.4 572.8 571.4 571.5 571.6

Autoimmune disease tissue 136.1 359.79 359.71 443.1 446 555 695.4 710 714 720 725 726 556 556.1 556.2 556.3 556.4 556.5 556.6 556.8 556.9 714.1 714.2 714.3 714.31

Gastrointestinal disease 531 531.2 531.4 531.6 532 532.2 532.4 532.6 533 533.2 533.4 533.6 534 534.2 534.4 534.6 535.01 535.11 535.21 535.31 535.41 535.51 535.61 535.71 562.02 562.03 562.12 562.13 569.3 569.85 569.86 578 578.1 578.9

Acute myocardial infarction 410 410.01 410.02 410.1 410.11 410.12 410.2 410.21 410.22 410.3 410.31 410.32 410.4 410.41 410.42 410.5 410.51 410.52 410.6 410.61 410.62 410.7 410.71 410.72 410.8

Peripheral vascular disease 250.7 443.9 443 443.1 443.2 443.21 443.22 443.23 443.24 443.29 443.8 443.81 443.82 443.89 441 443.9 785.4 V43.4

Chronic obstructive pulmonary disease 490 491 492 493 494 495 496 491.1 491.2 491.21 491.22 491.8 491.9 492.8 493.01 493.02 493.1 493.11 493.12 493.2 493.21 493.22 493.8 493.81 493.82

Renal diseases 582 582 582.1 582.2 582.4 582.8 582.81 582.89 582.9 583 583 583.1 583.2 583.4 583.6 583.7 585 585.1 585.2 585.3 585.4 585.5 585.6 585.9 586

Sleep disorders 327 327 327.01 327.02 327.09 327.1 327.1 327.11 327.12 327.13 327.14 327.15 327.19 327.2 327.2 327.21 327.22 327.23 327.24 327.25 327.26 327.27 327.29 327.3 327.3 327.31 327.32 327.33 327.34 327.35 327.36 327.37 327.39 327.4 327.4 327.41 327.42 327.43 327.44 327.49 327.5 327.51 327.52 327.53 327.59 327.8 780.51 780.53 780.57 307.45 307.4 780.5

Stroke/transient ischemic attack 430 431 433.91 434.11 434.91 435 435.1 435.2 435.3 435.8 435.9 436 438 438 438.1 438.1 438.11 438.12 438.13 438.14 438.19 438.2 438.2 438.21 438.22

Atrial fibrillation 427.31 429.4

Anemia 280 280.1 280.8 280.9 281 281.2 281.3 282 282.2 282.5 282.6 283 283.1 283.2 284 284.01 284.09 284.1 284.2 284.8 284.81 284.89 284.9 285 285.1

Cancer 140.x-209.x

**Supplementary Table 3. Baseline and clinical characteristics of patients with/without new onset depression before and after propensity score matching (1:1).**

* for SMD$\geq$0.2; SD: standard deviation; SGLT2I: sodium glucose cotransporter-2 inhibitor; DPP4I: dipeptidyl peptidase-4 inhibitor.

| **Characteristics** | **Before matching** |  | **SMD** | **After matching** |  | **SMD** |
| --- | --- | --- | --- | --- | --- | --- |
|  | **Depression (N=1113) Mean(SD);N or Count(%)** | **No depression (N=57393) Mean(SD);N or Count(%)** |  | **Depression (N=753) Mean(SD);N or Count(%)** | **No depression (N=38009) Mean(SD);N or Count(%)** |  |
| Demographics |  |  |  |  |  |  |
| Male gender | 367(32.97%) | 32458(56.55%) | 0.49* | 280(37.18%) | 23822(62.67%) | 0.53* |
| Female gender | 746(67.02%) | 24935(43.44%) | 0.49* | 473(62.81%) | 14187(37.32%) | 0.53* |
| Baseline age, years | 69.8(14.4);n=1113 | 63.3(12.8);n=57393 | 0.48* | 59.3(12.0);n=753 | 58.1(11.2);n=38009 | 0.11 |
| Past comorbidities |  |  |  |  |  |  |
| Charlson comorbidity index | 2.9(1.8);n=1113 | 2.1(1.5);n=57393 | 0.5* | 1.7(1.3);n=753 | 1.6(1.2);n=38009 | 0.1 |
| Heart failure | 67(6.01%) | 1872(3.26%) | 0.13 | 18(2.39%) | 950(2.49%) | 0.01 |
| Hypertension | 418(37.55%) | 13506(23.53%) | 0.31* | 237(31.47%) | 8628(22.69%) | 0.2 |
| Hypoglycemia | 24(2.15%) | 452(0.78%) | 0.11 | 7(0.92%) | 83(0.21%) | 0.09 |
| Hyperlipidaemia | 42(3.77%) | 1517(2.64%) | 0.06 | 31(4.11%) | 1313(3.45%) | 0.03 |
| Ischemic heart disease | 137(12.30%) | 5686(9.90%) | 0.08 | 91(12.08%) | 4519(11.88%) | 0.01 |
| Liver diseases | 59(5.30%) | 2151(3.74%) | 0.07 | 42(5.57%) | 1704(4.48%) | 0.05 |
| Autoimmune disease tissue | 24(2.15%) | 567(0.98%) | 0.09 | 10(1.32%) | 367(0.96%) | 0.03 |
| Gastrointestinal disease | 54(4.85%) | 1324(2.30%) | 0.14 | 18(2.39%) | 670(1.76%) | 0.04 |
| Acute myocardial infarction | 32(2.87%) | 1566(2.72%) | 0.01 | 21(2.78%) | 1290(3.39%) | 0.03 |
| Peripheral vascular disease | 10(0.89%) | 445(0.77%) | 0.01 | 3(0.39%) | 199(0.52%) | 0.02 |
| Chronic obstructive pulmonary disease | 26(2.33%) | 658(1.14%) | 0.09 | 14(1.85%) | 264(0.69%) | 0.1 |
| Renal diseases | 41(3.68%) | 1111(1.93%) | 0.11 | 3(0.39%) | 201(0.52%) | 0.02 |
| Sleep disorders | 37(3.32%) | 1712(2.98%) | 0.02 | 31(4.11%) | 1877(4.93%) | 0.04 |
| Stroke/transient ischemic attack | 65(5.84%) | 1789(3.11%) | 0.13 | 23(3.05%) | 947(2.49%) | 0.03 |
| Atrial fibrillation | 53(4.76%) | 1470(2.56%) | 0.12 | 20(2.65%) | 825(2.17%) | 0.03 |
| Anemia | 111(9.97%) | 2351(4.09%) | 0.23* | 33(4.38%) | 814(2.14%) | 0.13 |
| Cancer | 38(3.41%) | 1591(2.77%) | 0.04 | 12(1.59%) | 768(2.02%) | 0.03 |
| Duration from earliest diabetes mellitus diagnosis date to baseline date, day | 551.7(1391.3);n=1113 | 499.7(1312.8);n=57393 | 0.04 | 361.9(1014.3);n=753 | 461.7(1191.7);n=38009 | 0.09 |
| Medications |  |  |  |  |  |  |
| SGLT2I v.s. DPP4I | 200(17.96%) | 19181(33.42%) | 0.36* | 200(26.56%) | 19181(50.46%) | 0.51* |
| SGLT2I frequency | 11.6(17.4);n=200 | 7.0(9.5);n=19181 | 0.33* | 11.6(17.4);n=200 | 7.0(9.5);n=19181 | 0.33* |
| DPP4I frequency | 6.0(9.9);n=913 | 5.2(7.3);n=38212 | 0.09 | 6.3(13.0);n=553 | 3.9(7.0);n=18828 | 0.23* |
| SGLT2I duration, days | 627.1(696.8);n=200 | 523.4(670.0);n=19181 | 0.15 | 627.1(696.8);n=200 | 523.4(670.0);n=19181 | 0.15 |
| DPP4I duration, days | 478.9(287.6);n=913 | 505.9(285.5);n=38212 | 0.09 | 480.1(270.6);n=553 | 479.6(290.3);n=18828 | <0.01 |
| Metformin | 927(83.28%) | 50897(88.68%) | 0.16 | 710(94.28%) | 35347(92.99%) | 0.05 |
| Sulphonylurea | 871(78.25%) | 44112(76.85%) | 0.03 | 593(78.75%) | 27251(71.69%) | 0.16 |
| Insulin | 798(71.69%) | 28639(49.89%) | 0.46* | 461(61.22%) | 20234(53.23%) | 0.16 |
| Acarbose | 31(2.78%) | 1439(2.50%) | 0.02 | 25(3.32%) | 1430(3.76%) | 0.02 |
| Thiozolidinedone | 174(15.63%) | 10584(18.44%) | 0.07 | 217(28.81%) | 9307(24.48%) | 0.1 |
| Glucagon-like peptide-1 receptor agonists | 20(1.79%) | 1552(2.70%) | 0.06 | 24(3.18%) | 2408(6.33%) | 0.15 |
| Statins and fibrates | 195(17.52%) | 15097(26.30%) | 0.21* | 83(11.02%) | 4434(11.66%) | 0.02 |
| ACEI/ARB | 220(19.76%) | 12200(21.25%) | 0.04 | 313(41.56%) | 15275(40.18%) | 0.03 |
| Antihypertensive drugs | 9(0.80%) | 1009(1.75%) | 0.08 | 29(3.85%) | 1198(3.15%) | 0.04 |
| Anticoagulants | 307(27.58%) | 17071(29.74%) | 0.05 | 485(64.40%) | 22593(59.44%) | 0.1 |
| Antiplatelets | 213(19.13%) | 8941(15.57%) | 0.09 | 290(38.51%) | 10962(28.84%) | 0.21* |
| Lipid-lowering drugs | 220(19.76%) | 10779(18.78%) | 0.02 | 250(33.20%) | 13625(35.84%) | 0.06 |
| Nitrates | 99(8.89%) | 4140(7.21%) | 0.06 | 100(13.28%) | 5112(13.44%) | <0.01 |
| Non-steroidal anti-inflammatory drugs | 210(18.86%) | 8586(14.96%) | 0.1 | 289(38.37%) | 10661(28.04%) | 0.22* |
| Diuretics | 230(20.66%) | 9237(16.09%) | 0.12 | 220(29.21%) | 10637(27.98%) | 0.03 |
| Beta-blockers | 167(15.00%) | 7126(12.41%) | 0.08 | 181(24.03%) | 8863(23.31%) | 0.02 |
| Calcium channel blockers | 261(23.45%) | 12345(21.50%) | 0.05 | 328(43.55%) | 14845(39.05%) | 0.09 |
| Calculated biomarkers |  |  |  |  |  |  |
| Neutrophil-to-lymphocyte ratio | 4.3(5.8);n=620 | 3.6(4.9);n=23319 | 0.12 | 3.4(3.9);n=450 | 3.2(4.2);n=16901 | 0.03 |
| Platelet-to-lymphocyte ratio | 154.9(102.1);n=620 | 146.5(155.7);n=23316 | 0.06 | 135.9(69.1);n=450 | 138.6(140.5);n=16899 | 0.02 |
| Prognostic nutritional index | 40.1(5.9);n=826 | 40.9(6.4);n=36770 | 0.14 | 41.6(3.88);n=569 | 41.59(6.04);n=26243 | <0.01 |
| Complete blood counts |  |  |  |  |  |  |
| Mean corpuscular volume, fL | 87.7(7.5);n=733 | 87.2(7.6);n=28971 | 0.06 | 86.8(5.8);n=528 | 86.7(7.5);n=20632 | 0.01 |
| Eosinophil, x10^9/L | 0.2(0.21);n=621 | 0.22(0.26);n=23295 | 0.08 | 0.2(0.22);n=451 | 0.23(0.24);n=16894 | 0.09 |
| Lymphocyte, x10^9/L | 1.9(0.8);n=621 | 2.0(0.9);n=23320 | 0.09 | 2.0(0.8);n=451 | 2.1(0.9);n=16902 | 0.15 |
| Neutrophil, x10^9/L | 5.7(3.2);n=621 | 5.3(2.8);n=23320 | 0.13 | 5.28(2.47);n=451 | 5.29(2.84);n=16902 | 0.01 |
| White cell count, x10^9/L | 8.3(3.2);n=733 | 8.0(3.0);n=28980 | 0.11 | 7.8(2.6);n=528 | 8.1(3.1);n=20649 | 0.08 |
| Mean cell haemoglobin, pg | 29.6(3.0);n=733 | 29.4(3.0);n=28971 | 0.06 | 29.4(2.6);n=528 | 29.2(3.0);n=20632 | 0.07 |
| Platelet, x10^9/L | 241.8(73.5);n=733 | 239.2(72.4);n=28978 | 0.04 | 239.2(72.5);n=528 | 245.7(72.6);n=20647 | 0.09 |
| Red cell count, x10^12/L | 4.3(0.7);n=733 | 4.5(0.7);n=28971 | 0.36* | 4.5(0.6);n=528 | 4.7(0.7);n=20632 | 0.29* |
| Liver and renal functions |  |  |  |  |  |  |
| Potassium, mmol/L | 4.3(0.5);n=963 | 4.4(0.5);n=47431 | 0.16 | 4.2(0.4);n=662 | 4.3(0.5);n=31134 | 0.15 |
| Albumin, g/L | 40.4(4.4);n=818 | 41.6(4.0);n=36218 | 0.27* | 41.7(3.5);n=568 | 42.1(3.9);n=25915 | 0.12 |
| Sodium, mmol/L | 139.2(3.5);n=963 | 139.3(3.0);n=47456 | 0.03 | 138.4(3.5);n=662 | 139.3(2.8);n=31162 | 0.27* |
| Urea, mmol/L | 7.2(4.2);n=963 | 6.7(3.6);n=47440 | 0.12 | 6.6(4.8);n=676 | 6.2(2.8);n=31153 | 0.11 |
| Protein, g/L | 73.5(6.2);n=781 | 73.8(5.6);n=34024 | 0.05 | 75.4(7.1);n=554 | 74.3(5.4);n=24660 | 0.18 |
| Creatinine, umol/L | 104.6(92.9);n=964 | 97.0(79.9);n=47581 | 0.09 | 95.8(65.8);n=676 | 87.7(53.6);n=31207 | 0.14 |
| Alkaline phosphatase, U/L | 82.0(29.8);n=821 | 77.1(33.0);n=36335 | 0.15 | 81.5(27.9);n=568 | 76.5(30.6);n=25973 | 0.17 |
| Aspartate transaminase, U/L | 24.5(29.8);n=357 | 28.3(55.1);n=14402 | 0.09 | 27.0(30.4);n=336 | 29.5(33.5);n=10930 | 0.08 |
| Alanine transaminase, U/L | 23.1(17.1);n=658 | 28.9(34.6);n=30926 | 0.21* | 31.8(22.9);n=434 | 31.5(28.4);n=21599 | 0.01 |
| Bilirubin, umol/L | 9.8(5.2);n=817 | 11.3(7.0);n=36147 | 0.25* | 9.7(4.5);n=568 | 11.3(6.0);n=25869 | 0.29* |
| Lipid and glucose profiles |  |  |  |  |  |  |
| Triglyceride, mmol/L | 1.8(1.7);n=844 | 1.7(1.5);n=44681 | 0.08 | 1.9(1.8);n=616 | 1.8(1.6);n=29550 | 0.04 |
| SD of triglyceride | 0.52(0.83);n=424 | 0.47(0.94);n=22877 | 0.06 | 0.7(1.1);n=361 | 0.5(1.0);n=17460 | 0.18 |
| Low-density lipoprotein, mmol/L | 2.36(0.84);n=831 | 2.38(0.8);n=43974 | 0.02 | 2.45(0.81);n=613 | 2.4(0.82);n=29025 | 0.06 |
| SD of low-density lipoprotein | 0.43(0.4);n=416 | 0.36(0.34);n=22283 | 0.17 | 0.5(0.4);n=364 | 0.4(0.3);n=16974 | 0.27* |
| High-density lipoprotein, mmol/L | 1.23(0.37);n=842 | 1.2(0.33);n=44623 | 0.09 | 1.3(0.4);n=614 | 1.2(0.3);n=29502 | 0.23* |
| SD of high-density lipoprotein | 0.12(0.1);n=415 | 0.1(0.08);n=22010 | 0.19 | 0.12(0.09);n=361 | 0.1(0.08);n=16749 | 0.28* |
| Total cholesterol, mmol/L | 4.4(1.1);n=845 | 4.3(1.0);n=44725 | 0.07 | 4.5(1.1);n=616 | 4.4(1.0);n=29576 | 0.17 |
| SD of total cholesterol | 0.5(0.4);n=425 | 0.4(0.4);n=22895 | 0.13 | 0.5(0.4);n=364 | 0.4(0.4);n=17484 | 0.21* |
| Hemoglobin A1C, % | 7.98(1.64);n=920 | 8.02(1.53);n=46664 | 0.03 | 8.7(2.3);n=651 | 8.2(1.6);n=30667 | 0.26* |
| SD of hemoglobin A1C | 0.7(0.7);n=592 | 0.6(0.7);n=32775 | 0.19 | 1.0(1.0);n=458 | 0.6(0.8);n=23291 | 0.46* |
| Fasting glucose, mmol/L.1 | 9.5(4.9);n=834 | 8.9(3.9);n=42172 | 0.15 | 10.1(5.5);n=573 | 9.1(4.4);n=27559 | 0.19 |
| SD of fasting glucose | 2.7(2.6);n=548 | 2.0(2.2);n=26158 | 0.29* | 2.7(2.7);n=396 | 2.0(2.2);n=19598 | 0.3* |

**Supplementary Table 3. Univariate Cox regression models to predict new onset depression, new onset anxiety disorder, and mortality risk in patients with type-2 diabetes mellitus in the matched and unmatched cohorts.**

* for p≤ 0.05, ** for p ≤ 0.01, *** for p ≤ 0.001; HR: hazard ratio; CI: confidence interval; SD: standard deviation; SGLT2I: sodium glucose cotransporter-2 inhibitor; DPP4I: dipeptidyl peptidase-4 inhibitor.

| **Characteristics** | **Before Matching** | **After Matching** |
| --- | --- | --- |
|  | **Depression HR [95% CI];P value** | **Depression HR [95% CI];P value** |
| ***Demographics*** |  |  |
| Male gender | 1.0[Reference] | 1.0[Reference] |
| Female gender | 2.63[2.32-2.98];<0.0001*** | 2.80[2.41-3.24];<0.0001*** |
| Baseline age, years | 1.05[1.04-1.05];<0.0001*** | 1.01[1.00-1.02];0.0010** |
| ***Past comorbidities*** |  |  |
| Charlson comorbidity index | 1.33[1.29-1.37];<0.0001*** | 1.09[1.04-1.15];0.0012** |
| Heart failure | 2.03[1.59-2.60];<0.0001*** | 1.00[0.62-1.59];0.9833 |
| Hypertension | 1.98[1.76-2.24];<0.0001*** | 1.56[1.34-1.82];<0.0001*** |
| Hypoglycaemia | 2.91[1.94-4.35];<0.0001*** | 4.27[2.03-8.98];0.0001*** |
| Hyperlipidaemia | 1.44[1.06-1.96];0.0198* | 1.20[0.84-1.72];0.3216 |
| Ischemic heart disease | 1.29[1.08-1.55];0.0048** | 1.02[0.82-1.28];0.8256 |
| Liver diseases | 1.44[1.11-1.87];0.0063** | 1.26[0.92-1.72];0.1497 |
| Autoimmune disease | 2.17[1.45-3.26];0.0002*** | 1.38[0.74-2.57];0.3136 |
| Gastrointestinal disease | 2.20[1.68-2.90];<0.0001*** | 1.37[0.86-2.18];0.1917 |
| Acute myocardial infarction | 1.09[0.76-1.54];0.6457 | 0.83[0.54-1.28];0.4038 |
| Peripheral vascular disease | 1.25[0.67-2.33];0.4838 | 0.80[0.26-2.47];0.6923 |
| Chronic obstructive pulmonary disease | 2.17[1.47-3.20];0.0001*** | 2.70[1.59-4.58];0.0002*** |
| Renal diseases | 2.08[1.52-2.84];<0.0001*** | 0.76[0.24-2.36];0.6340 |
| Sleep disorders | 1.10[0.79-1.53];0.5715 | 0.82[0.57-1.18];0.2822 |
| Stroke/transient ischemic attack | 1.97[1.53-2.53];<0.0001*** | 1.25[0.82-1.89];0.2950 |
| Atrial fibrillation | 1.99[1.51-2.62];<0.0001*** | 1.25[0.80-1.96];0.3174 |
| Anaemia | 2.69[2.21-3.28];<0.0001*** | 2.10[1.48-2.98];<0.0001*** |
| Cancer | 1.28[0.93-1.78];0.1290 | 0.81[0.46-1.42];0.4575 |
| ***Medications*** |  |  |
| SGLT2I v.s. DPP4I | 0.42[0.36-0.49];<0.0001*** | 0.35[0.30-0.41];<0.0001*** |
| Metformin | 0.60[0.51-0.71];<0.0001*** | 1.22[0.90-1.66];0.1999 |
| Sulphonylurea | 1.08[0.94-1.25];0.2808 | 1.46[1.23-1.74];<0.0001*** |
| Insulin | 2.61[2.29-2.98];<0.0001*** | 1.40[1.21-1.63];<0.0001*** |
| Acarbose | 1.11[0.78-1.59];0.5511 | 0.88[0.59-1.31];0.5305 |
| Thiazolidinedione | 0.80[0.68-0.94];0.0074** | 1.23[1.05-1.44];0.0101* |
| Glucagon-like peptide-1 receptor agonists | 0.64[0.41-1.00];0.0516 | 0.48[0.32-0.73];0.0005*** |
| Statins and fibrates | 0.59[0.51-0.69];<0.0001*** | 0.93[0.74-1.17];0.5584 |
| ACEI/ARB | 0.91[0.79-1.06];0.2301 | 1.07[0.92-1.23];0.3845 |
| Antihypertensive drugs | 0.45[0.23-0.87];0.0176* | 1.23[0.85-1.78];0.2813 |
| Anticoagulants | 0.90[0.79-1.03];0.1248 | 1.24[1.07-1.44];0.0046** |
| Antiplatelets | 1.29[1.11-1.49];0.0009*** | 1.55[1.34-1.80];<0.0001*** |
| Lipid-lowering drugs | 1.07[0.92-1.24];0.3941 | 0.89[0.77-1.04];0.1351 |
| Nitrates | 1.27[1.03-1.56];0.0246* | 1.00[0.81-1.23];0.9640 |
| Non-steroidal anti-inflammatory drugs | 1.33[1.14-1.54];0.0002*** | 1.60[1.38-1.86];<0.0001*** |
| Diuretics | 1.38[1.19-1.59];<0.0001*** | 1.08[0.92-1.26];0.3501 |
| Beta-blockers | 1.25[1.06-1.47];0.0081** | 1.05[0.89-1.24];0.5708 |
| Calcium channel blockers | 1.12[0.98-1.29];0.1081 | 1.21[1.05-1.40];0.0099** |
| ***Complete blood counts*** |  |  |
| Mean corpuscular volume, fL | 1.01[1.00-1.02];0.0728 | 1.00[0.99-1.01];0.8268 |
| Eosinophil, x10^9/L | 0.66[0.43-1.00];0.0500 | 0.60[0.36-0.99];0.0459* |
| Lymphocyte, x10^9/L | 0.87[0.78-0.96];0.0063** | 0.82[0.73-0.93];0.0018** |
| Neutrophil, x10^9/L | 1.04[1.02-1.06];0.0002*** | 1.00[0.97-1.03];0.9911 |
| White cell count, x10^9/L | 1.02[1.01-1.03];0.0009*** | 0.97[0.94-1.01];0.0959 |
| Mean cell haemoglobin, pg | 1.03[1.00-1.05];0.0542 | 1.02[1.00-1.06];0.1026 |
| Platelet, x10^9/L | 1.000[0.999-1.001];0.4266 | 0.999[0.997-1.000];0.0342* |
| Red cell count, x10^12/L | 0.58[0.52-0.64];<0.0001*** | 0.64[0.56-0.73];<0.0001*** |
| ***Liver and renal functions*** |  |  |
| Potassium, mmol/L | 0.71[0.62-0.81];<0.0001*** | 0.72[0.61-0.86];0.0002*** |
| Albumin, g/L | 0.93[0.92-0.95];<0.0001*** | 0.97[0.95-0.99];0.0029** |
| Sodium, mmol/L | 0.99[0.97-1.01];0.2544 | 0.91[0.89-0.93];<0.0001*** |
| Urea, mmol/L | 1.03[1.02-1.05];<0.0001*** | 1.04[1.02-1.06];<0.0001*** |
| Protein, g/L | 0.99[0.98-1.00];0.0838 | 1.04[1.02-1.06];<0.0001*** |
| Creatinine, umol/L | 1.001[1.000-1.002];0.0005*** | 1.002[1.001-1.002];0.0001*** |
| Alkaline phosphatase, U/L | 1.003[1.002-1.004];<0.0001*** | 1.00[1.00-1.01];<0.0001*** |
| Aspartate transaminase, U/L | 0.99[0.99-1.00];0.0678 | 1.00[0.99-1.00];0.1654 |
| Alanine transaminase, U/L | 0.98[0.98-0.99];<0.0001*** | 1.000[0.997-1.004];0.8147 |
| Bilirubin, umol/L | 0.94[0.93-0.96];<0.0001*** | 0.94[0.92-0.96];<0.0001*** |
| ***Lipid and glucose profiles*** |  |  |
| Triglyceride, mmol/L | 1.04[1.01-1.06];0.0081** | 1.02[0.98-1.06];0.3398 |
| Low-density lipoprotein, mmol/L | 0.97[0.89-1.06];0.5632 | 1.08[0.98-1.18];0.1187 |
| High-density lipoprotein, mmol/L | 1.31[1.08-1.59];0.0059** | 1.75[1.49-2.05];<0.0001*** |
| Total cholesterol, mmol/L | 1.07[1.01-1.14];0.0338* | 1.16[1.08-1.23];<0.0001*** |
| SD of total cholesterol | 1.30[1.09-1.56];0.0044** | 1.44[1.22-1.69];<0.0001*** |
| Haemoglobin A1C, % | 0.98[0.94-1.02];0.3687 | 1.12[1.09-1.14];<0.0001*** |
| Fasting glucose, mmol/L.1 | 1.03[1.02-1.04];<0.0001*** | 1.03[1.02-1.04];<0.0001*** |
| SD of fasting glucose | 1.10[1.07-1.12];<0.0001*** | 1.10[1.07-1.14];<0.0001*** |
